# Cutoff points of adiposity anthropometric indices for low muscle mass screening in middle-aged and older healthy women

**DOI:** 10.1186/s12891-021-04532-x

**Published:** 2021-08-20

**Authors:** Rafaela Andrade do Nascimento, Mariana Carmem Apolinário Vieira, Rafaella Silva dos Santos Aguiar Gonçalves, Mayle Andrade Moreira, Maria Socorro Medeiros de Morais, Saionara Maria Aires da Câmara, Álvaro Campos Cavalcanti Maciel

**Affiliations:** 1 Physiotherapy, Department of Federal, University of Rio Grande Do Norte, 3000 Senador Salgado Filho Avenue, S/N, Lagoa Nova, Natal, RN CEP: 59072-970 Brazil; 2 Physiotherapy Department of Federal, University of Ceará, 949 Alexandre Baraúna St, Rodolfo Teófilo, Fortaleza, CEP: 60430-110 Brazil; 3 Health Sciences Center of Federal, University of Rio Grande Do Norte, General Gustavo Cordeiro de Farias St, Petrópolis, Natal, RN CEP 59012-570 Brazil; 4 Faculty of Health Sciences of Trairi, Santa Cruz, Brazil, Vila Trairi St, Santa Cruz, RN CEP: 59200-000 Brazil

**Keywords:** Aging, Body composition, Adiposity, Muscle mass, Women

## Abstract

**Background:**

The reduction of female sex hormones causes changes in the contractile properties of muscles as well as infiltration of fat in the muscle tissue. This results in a consequent decline in muscle strength. These changes are related to higher levels of functional impairment and physical disability. In this sense, several anthropometric indices have been used to quantify body and visceral fat. Thus, the objective of this paper is to propose cutoff points for adiposity anthropometric indices in order to identify low muscle mass, as well as to analyze the relationship between these indices and low muscle mass in middle-aged and older women.

**Methods:**

Cross-sectional analytical study carried out in the Northeast of Brazil. The sample was formed by 593 women between 40—80 years old. Data collection included anthropometric assessment (BMI: Body Mass Index – WC: Waist Circumference – WHR: Waist-to-hip Ratio – WHtR: Waist-to-height Ratio – CI: Conicity Index – BAI: Body Adiposity Index – VAI: Visceral Adiposity Index – LAP: Lipid Accumulation Product), bioimpedance test and biochemical dosage. Moreover, sociodemographic data and practice of physical activity were collected. Descriptive statistics, Student's t-test, ROC curves, chi-squared and logistic regression were performed.

**Results:**

The participants had a mean age of 53.11 (8.89) years, BMI of 28.49 (5.17) kg/m^2^ and WC of 95.35 (10.39). The prevalence of low muscle mass was 19.4%. Based on sensitivity and specificity of adiposity anthropometric indices, cutoff points were developed to identify the presence of low muscle mass (*p* < 0.05), except for VAI. After logistic regression, WC (OR = 6.2; CI 95%: 1.4—28.1), WHR (OR = 1.8; CI: 1.0—3.4), WHtR (OR = 5.0; CI 95%: 1.0—23.7) and BAI (OR = 14.5; CI 95%: 6.6—31.7) were associated with low muscle mass.

**Conclusions:**

All anthropometric indices, except VAI, showed adequate accuracy in identifying low muscle mass in women, especially those that took into account WC. This suggests that they can become accessible and also be cost-effective strategies for assessing and managing health outcomes related to muscle mass analysis.

## Background

Female aging is mainly characterized by a decrease in the levels of sex hormones, which culminates in menopause and causes important changes in body composition and the musculoskeletal system [1, 2]. Among the main biological changes are the decline in skeletal muscle mass and bone mass, in addition to the increase of fat mass [1, 3–6].

In women, from middle age onward, acceleration in the loss of muscle mass and strength is common, which is not observed in men in the same proportion [2, 7], pointing to a strong correlation between skeletal muscle mass and estradiol levels [8]. There are also changes in the contractile properties of muscles and infiltration of fat into the tissue, with a consequent decline in muscle strength [1, 9]. Thus, low serum estrogen in the postmenopausal period is the main cause of the rapid decline in muscle strength and physical performance observed in older women [10].

Regarding fat mass, it is known that its excess in the body, known as adiposity, is a well-established risk factor for worse health outcomes as it is an active contributor to changes in metabolic profiles [6]. In addition, there is change in the pattern of distribution of body fat, as it is deposited more in the abdominal region, contributing to the weight gain characteristic of the menopausal transition [11].

It is known that impaired muscle strength and increased visceral adipose tissue appear to be associated with worse long-term survival [12]. Considering that obesity is the main modifiable risk factor for diabetes, cardiovascular disease, dyslipidemia and hypertension, and that abdominal obesity has been linked to all-cause obesity-related mortality and mortality, one of the best clinical uses of anthropometric data is define obesity [13].

In this perspective, several anthropometric indices have been used to quantify body and visceral fat [14–16]. These measures have been shown to be effective in anthropometric assessment in several epidemiological studies as they are simple, safe, low-cost and clinically useful, especially when more sophisticated and costly resources are not available [17].

Among the most known anthropometric indices are the body mass index (BMI), waist circumference (WC), waist-to-hip ratio (WHR) and waist-to-height ratio (WHtR) [18]. However, other indices such as those that employ anthropometry using formulas have also been applied, highlighting the conicity Index (CI) (central adiposity) [16] and the body adiposity index (BAI) (body adiposity) [14], the visceral adiposity index (VAI) and the lipid accumulation product (LAP), both of which are markers of central adiposity [19].

All these bodily changes that occur with female aging are related to higher levels of fragility, functional impairment and disability [20], since the loss of muscle mass and strength starts earlier in women. Moreover, this group also appears to be subject to sarcopenia at an earlier age [7]. Several studies have shown that anthropometric measurements are useful for tracking sarcopenia associated with conditions of reduced muscle mass, falls, functionality and mortality [21, 22].

It is well established that there are significant associations between low muscle mass, changes in body composition and increased mortality [23, 24]. Still, regarding anthropometric indices, it can be observed that research carried out is punctual, involving one or more anthropometric indices and their relationship with cardiometabolic risk, diseases and functionality [7, 15, 18].

Given the scarcity of studies that validate anthropometric measures as instruments capable of identifying sarcopenia in the elderly, it is believed that the indicators may represent a viable and additional alternative to be used to facilitate screening, in order to guide the diagnosis of sarcopenia and the adequate interventions, with an impact on the health care of the elderly population [25, 26].

In addition, considering that changes in body composition in women start during middle age, a period in which menopause usually occurs, it is of fundamental importance to explore these processes both during aging and in phases immediately before their onset. Therefore, the present study aimed to identify cutoff points that detect low muscle mass according to anthropometric indices used, as well as to analyze the relationship between these indices and the presence of low muscle mass in middle-aged and older women.

## Materials and methods

### Participants

This is a cross-sectional, observational and analytical study carried out in two cities in the Northeast of Brazil (Parnamirim and Santa Cruz). Additional information about the cities can be obtained in other studies [7, 11].

The study population consisted of women aged 40 to 80 years. To be eligible for the study, participants should meet the following inclusion criteria be clinically healthy at the time of the interview, have not undergone bilateral oopherectomy or hysterectomy, and do not have neurological diseases or other conditions that could compromise any stage of data assessment.

The project was advertised in the Basic Health Units of the municipalities, with the sample formed by convenience, from the women who participated in the health examinations of the Menopausa Saudável Project [7, 11]. In these studies, 708 participants were evaluated, of which, for the present study, 115 were excluded because they did not present all the necessary data related to biochemical tests or were part of other studies in the research group, totaling 593 women.

### Ethical aspects

This research study received ethics approval by the Ethics and Research Committee of the Federal University of Rio Grande do Norte (approval number 1.875.802). All procedures in this study were in accordance with the code of ethics of the World Medical Association (declaration of Helsinki). All participants were informed of the objectives and procedures of the research at a first contact and an informed consent was obtained.

### Procedures

Participants were evaluated by trained physical therapists using standardized protocols and blood samples were collected by trained nurse technicians. The evaluation protocol included anthropometric evaluation, electrical bioimpedance for analysis of muscle mass, biochemical dosage, in addition to the collection of sociodemographic data and on the practice of physical activity, as described below.

### Anthropometric adiposity indices

- Body Mass Index (BMI)
  BMI (kg/m^2^) was calculated from the measurement of height (m) and weight (kg) [7]. Weight measurement (kg) was obtained using a Wiso® digital scale, model W903. Height (m) was recorded using a Welmy® stadiometer.
- Waist Circumference (WC), Hip Circumference (HC), Waist-to-hip ratio (WHR) and waist-to- height ratio (WHtR)
  For measurements of waist circumference (cm) and hips (cm), a “fiber glass” measuring tape was used, with divisions of 1 mm. WC was measured at the midpoint between the iliac crest and the last rib, and HC was measured at the most prominent area of the buttocks [27]. For the WHR calculation, the waist circumference measurement value was divided by the hip circumference value. For the WHtR calculation, the waist circumference value was divided by height [28]. In the case of WtHR, height was measured in centimeters [16].
- Conicity Index (CI)
  CI was calculated using the equation (CI = WC (m) / [0.109 x √ {weight (kg) / height (m)}, where 0.109 is a constant that results from converting units of volume and mass into units of length [15].
- Body Adiposity index (BAI), Visceral Adiposity Index (VAI) and Lipidic Accumulation Product (LAP)

Measurements were calculated according to previous studies found in literature [14–16]. Triglycerides (TG) and High-Density Lipoprotein (HDL) measurements presented in the formulas below were expressed in mmol/l (millimol per liter) [16].

$$\begin{array}{*{20}l} {{\text{BAI}}:\left( {{{{\text{WC}}\left( {{\text{cm}}} \right)} \mathord{\left/ {\vphantom {{{\text{WC}}\left( {{\text{cm}}} \right)} {{\text{HEIGHT}}\left( {\text{m}} \right)^{{{1},{5}}} }}} \right. \kern-\nulldelimiterspace} {{\text{HEIGHT}}\left( {\text{m}} \right)^{{{1},{5}}} }}} \right) - {18}} \hfill \\ {{\text{VAI}}:\left[ {{{{\text{WC}}\left( {{\text{cm}}} \right)} \mathord{\left/ {\vphantom {{{\text{WC}}\left( {{\text{cm}}} \right)} {{36}.{58} + \left( {{1}.{89} \times {\text{BMI}}} \right)}}} \right. \kern-\nulldelimiterspace} {{36}.{58} + \left( {{1}.{89} \times {\text{BMI}}} \right)}}} \right] \times \left( {{{{\text{TG}}} \mathord{\left/ {\vphantom {{{\text{TG}}} {0.{81}}}} \right. \kern-\nulldelimiterspace} {0.{81}}}} \right) \times \left( {{{{1}.{52}} \mathord{\left/ {\vphantom {{{1}.{52}} {{\text{HDL}}}}} \right. \kern-\nulldelimiterspace} {{\text{HDL}}}}} \right)} \hfill \\ {{\text{LAP}}:\left( {{\text{WC}}\left( {{\text{cm}}} \right){-}{58}} \right) \times {\text{TG}}} \hfill \\ \end{array}$$

### Low muscle mass

Skeletal muscle mass was calculated using electrical bioimpedance analysis, with a portable body mass analyzer Inbody R20, using the manufacturer’s prediction equations [29]. Bioimpedance shows results of muscle mass for each limb of a person. The measure of skeletal muscle mass used was the Appendicular Skeletal Muscle (ASM). This was defined by the sum of the muscle mass of the four limbs in kilograms. Then, the result for each participant was normalized by the measured height expressed in meters, using the following formula: ASM/height^2^ (kg/m2) [30]. The cutoff point established in the present study to detect low muscle mass was calculated by the 20th percentile of the sample [26], being 5.97 kg/m^2^. Although BIA equipment does not measure muscle mass directly as DEXA/MRI, it uses a conversion equation that is calibrated with a reference of DXA-measured lean mass [26, 29]. A recent systematic review [31] showed that BIA presents high concurrent validity with DEXA (AUC > 70), but they found that reliability data on BIA are lacking. Although it is not the gold standard measure for muscle mass, BIA is widely used and presents a good option for epidemiological research [26]. The assessment of muscle mass was performed by a blind evaluator. Considering the use of bioimpedance, the participants were advised to go use the toilet for urination or defecation before the test and to avoid exercise, meals or bathing before the test.

### Biochemical dosage

The women were instructed to attend the Hospital Maternidade Divino Amor (Parnamirim/RN) or the University Hospital Ana Bezerra (Santa Cruz/RN), according to the municipality they lived in, on a day and time previously scheduled, after a 12-h fast, when blood samples were collected by trained nurse technicians. The dosage of the biochemical parameters used, TG and HDL, were analyzed using the calorimetric enzymatic method by specialized laboratory technicians.

### Covariables

Sociodemographic data such as age, marital status, education and family income were collected using a structured questionnaire. Marital status was categorized as yes or no. Education was categorized into: less than Basic Education (up to 7 years), between Basic and Secondary Education (more than 7 and less than 11 years), and Secondary or more (11 + years). Family income was dichotomized in earning less than 3 minimum wages (MW) per month or 3 MW or more per month, according to the Brazilian minimum wage ate the time of the survey [7].

In addition, data regarding the practice of physical activity were also collected by self-report. The participants were asked about participation in sports, exercises or other physical activities, at least three times a week and for thirty minutes or more each time, being characterized as yes or no [7].

### Data analysis

Analyzes were performed using the SPSS program (Statistical Package for the Social Sciences), version 20.0 (SPSS, Chicago, IL, USA). Initially, the descriptive statistics of the variables were presented. Student’s t-test was used to compare the means between anthropometric indices and the presence or absence of low muscle mass.

The discriminatory capacity of anthropometric indices in the low muscle mass outcome variable was assessed by the area below the ROC curve. Sensitivity and specificity were then calculated to establish the cutoff points for anthropometric indices. Then, the chi-squared test (χ^2^) was performed to verify whether the established cutoff points were associated with low muscle mass.

Finally, a logistic regression analysis was performed to verify the effect of anthropometric indices on low muscle mass, adjusted for covariates that showed associations with *p* < 0.20 in the bivariate analysis and to calculate the Odds Ratio (OR) to measure the strength of the association found. A 95% confidence interval and a *P* value of 0.05 were adopted for statistical significance of data.

## Results

The sample consisted of 593 women, with a mean age of 53.11 (± 8.89) years. Most women studied until elementary school (45.2%) and had a family income of less than 3 minimum wages per month (71.0%). The prevalence of low muscle mass was 19.4%. The other characteristics of the sample, including body composition measures, are described in Table 1.

**Table 1:** Descriptive analysis (*n* = 593). Natal, RN, 2020

Variables	n (%) or Mean (SD)
Age (years)	53.11 (8.89)
Marital Status	
Yes	414 (69.8)
No	179 (30.2)
Education	
Less than basic education	268 (45.2)
Between basic and secondary	224 (37.8)
Secondary or more	101 (17.0)
Family income	
< 3 MW	421 (71.0)
≥ 3 MW	172 (29.0)
Physical activity	
Yes	187 (31.5)
No	406 (68.5)
TG	149.98 (72.02)
HDL	49.22 (13.55)
BMI (kg/m^2^)	28.49 (5.17)
WC (cm)	95.35 (10.39)
HC (cm)	104.67 (9.13)
WHR	0.92 (0.05)
WHtR	0.63 (0.07)
CI	1.33 (0.07)
BAI	37.61 (5.46)
VAI	7.48 (4.25)
LAP	68.46 (38.04)
Low muscle mass	
Yes	115 (19.4)
No	478 (80.6)

*Abbreviations*: *MW* Minimum Wage, *TG* Triglycerides, *HDL* High‐Density Lipoprotein, *BMI* Body Mass Index, *WC* Waist Circumference, *HC* Hip Circumference, *WHR* Waist-to-hip Ratio, *WHtR* Waist-to-height Ratio, *CI* Conicity Index, *BAI* Body Adiposity Index, *VAI* Visceral Adiposity Index, *LAP* Lipid Accumulation Product

The difference between the averages of anthropometric indices according to the presence or absence of muscle loss is described in Table 2. There were statistically significant differences for all variables analyzed, except for VAI only.

**Table 2:** Comparison of the values of anthropometric indices of adiposity according to the presence or absence of low muscle mass. Natal, RN, 2020

Variables	Low muscle mass		
	Yes (115)	No (478)	
	Mean (SD)	Mean (SD)	*P* value
BMI (kg/m^2^)	31.82 (5.34)	28.31 (4.42)	< 0.001
WC (cm)	100.23 (10.69)	94.68 (10.11)	< 0.001
WHR	0.93 (0.05)	0.90 (0.05)	0.001
WHtR	0.68 (0.06)	0.61 (0.06)	< 0.001
CI	1.35 (0.07)	1.31 (0.06)	< 0.001
BAI	42.79 (5.04)	36.28 (4.36)	< 0.001
VAI	7.61 (6.32)	8.09 (7.86)	0.51
LAP	73.38 (37.7)	63.36 (37.7)	0.02

*Abbreviations*: *BMI*, Body Mass Index; *WC*, Waist Circumference; *WHR*, Waist-to-hip Ratio; *WHtR*, Waist-to-height Ratio; *CI*, Conicity Index; *BAI*, Body Adiposity Index; *VAI*, Visceral Adiposity Index; *LAP*, Lipid Accumulation Product

*P* value < 0.05

Table 3 shows the areas under the ROC curve, cutoff points, sensitivity and specificity of body composition variables in relation to low muscle mass, as a way of identifying possible cutoff points that could discriminate between having low muscle mass or not. Thus, with the exception of VAI, statistically significant differences were observed for all variables that we analyzed.

**Table 3:** Area under the ROC curve (95% CI), cut-off point, sensitivity and specificity of anthropometric adiposity indices to identify low muscle mass. Natal, RN, 2020

Variables	Area under the curve (IC 95%)	Cut-off point	Sensitivity	Specificity	*P* value
BMI (kg/m^2^)	0.69 (0.63–0.75)	29.4	0.63	0.68	< 0.001
WC (cm)	0.65 (0.59–0.70)	94.3	0.53	0.75	< 0.001
WHR	0.61 (0.54–0.67)	0.91	0.54	0.67	0.001
WHtR	0.78 (0.73–0.83)	0.64	0.73	0.74	< 0.001
CI	0.64 (0.46- 0.75)	1.34	0.68	0.58	< 0.001
BAI	0.83 (0.79–0.88)	39.1	0.76	0.80	< 0.001
VAI	0.50 (0.44–0.56)	-	-	-	0.98
LAP	0.59 (0.54–0.65)	51.4	0.47	0.73	0.03

*Abbreviations*: *BMI* Body Mass Index, *WC* Waist Circumference, *WHR* Waist-to-hip Ratio, *WHtR* Waist-to-height Ratio, *CI* Conicity Index, *BAI* Body Adiposity Index, *VAI* Visceral Adiposity Index, *LAP* Lipid Accumulation Product

*P* value < 0.05

**Fig. 1:**
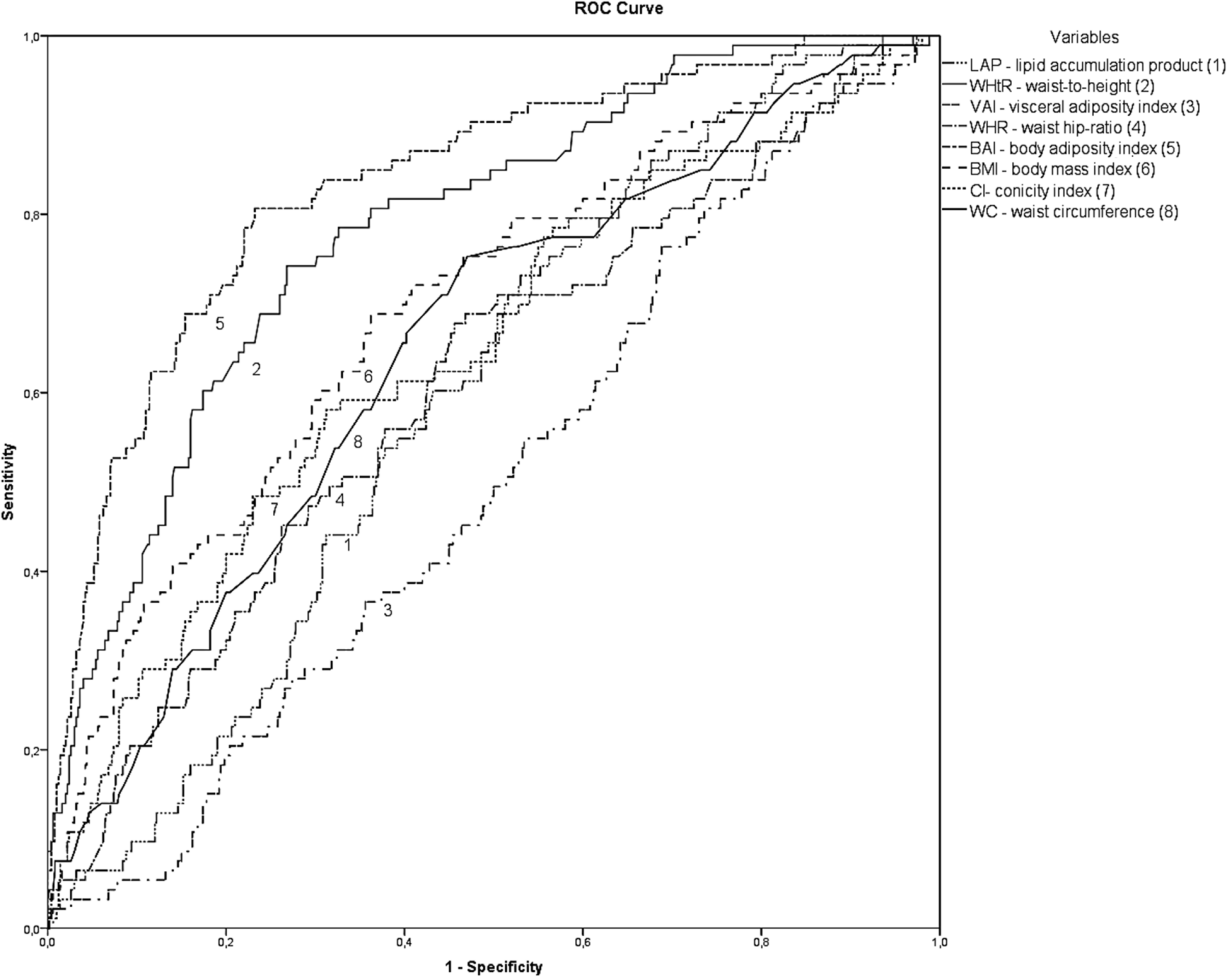
Area under the receiver operating characteristic curve of anthropometric indices for screening low muscle mass

Complementary, Fig. 1 shows the behavior of the ROC curves, where it is observed that BAI was the variable with the largest area under the curve, while VAI presented the smallest area under the curve.

Table 4 shows the results of the logistic regression, which was performed in order to analyze the power of association of anthropometric variables along with age, on low muscle mass. After the regression analyzes, Waist Circumference (> 94.3), Waist-to-height Ratio (> 0.64), Waist-to-hip Ratio (> 0.91), Body Adiposity Index (> 39.1) and age over 60 years were related to a greater risk of presenting low muscle mass.

**Table 4:** Logistic regression between anthropometric indices and low muscle mass according to covariates. Natal, 2020

Variables (*n* = 593)	OR	SE	Confidence interval (95%)	*P* value
Age (> 60 years)	3.8	0.29	2.2 – 6.8	0.001
WC (> 94.3)	6.2	0.77	1.4—28.1	0.001
WHR (> 0.91)	1.8	0.51	1.0 – 3.4	0.04
WHtR (> 0.64)	5.0	0.30	1.0 – 23.7	0.04
BAI (> 39.1)	14.5	0.39	6.6 – 31.7	0.001

*Abbreviations*: *OR* Odds Ratio, *SE* Standard Error, *WC* Waist Circumference, *WHR* Waist-to-hip Ratio, *WHtR* Waist-to-height Ratio, *BAI* Body Adiposity Index

*n* = 593

COVARIATES: marital status, education, family income and physical activity

## Discussion

As far as we know, this is one of the first studies that proposes to present cutoff points to define the presence of low muscle mass in middle-aged and older women. From the findings of the present study, different cutoff points for anthropometric adiposity indices were proposed to identify low muscle mass in a sample of middle-aged and older women. In addition, the cutoff points established were associated with the presence of low muscle mass after adjustments for confounding variables in logistic regression analysis.

It is known that muscle tissue contains lipid deposits found in the fascia, inside the muscle and also in muscle fibers, the latter being associated with insulin resistance, inflammation and functional deficit in skeletal muscle [32]. Our results demonstrate that women with low muscle mass had higher values in adiposity indices (BMI, BAI, WC, WHR, WHtR, CI and LAP) when compared to participants with adequate muscle mass. Our findings are in accordance with the current literature that has shown that obesity is not only related to adverse metabolic outcomes [33–35], but also negatively affects skeletal muscle [36, 37].

However, as previously mentioned, there is a lack of studies that have investigated the relationship between muscle mass with different adiposity rates, which makes comparability with our results difficult, since the most frequent adiposity rates in literature are BMI and WC measure [38, 39].

Our findings corroborate the study by Dabak et al. [40], which found lower values of muscle mass in obese women (BMI > 30 kg/m^2^). Also, in the *São Paulo Ageing & Health Study,* older women with low muscle mass also had higher BMI values and, in addition, in the multivariate analysis, the measure of visceral adiposity was an important predictor [41]. Nevertheless, in the study by Abramowitz et al. [42], it was observed that people with low muscle mass had lower BMI – 22.4 kg/m^2^ – than people with preserved muscle mass – 28.2 kg/m^2^. In this study, we found that a BMI < 29.4 was associated with having good muscle mass. This means that even having overweight, some women remain with good muscle mass, which highlights that the association between BMI and muscle mass may not be linear. Women with overweight need more muscle strength to move their body for daily activities, and previous studies have shown higher measures of muscle mass and strength associated with higher BMI [42, 43]. Thus, a certain degree of overweight may work as an overload that help to maintain or increase muscle mass. However, a greater accumulation of fat mass, as found in obese individuals, may promote the previously described deleterious effects on muscle tissue, leading to a reduction in the amount of muscle mass. Confirming these hypotheses, a previous study [42] found that, although overall people with low muscle mass had lower BMI, at a given level of BMI those with low muscle mass had higher percentage of total body fat. This reinforces the need of using different body fat measures to identify those at risk of presenting low muscle mass.

Regarding WC, the study by Siervo et al. [44], with women aged 18 years and older, reinforces our findings, demonstrating that regardless of age group (< 60 years or > 60 years), a waist circumference greater than 88 cm can better discriminate women with low muscle mass associated with high adiposity than a BMI greater than 30 kg/m^2^ [44].

A study carried out with obese and overweight older adults with metabolic syndrome, of both genders, found a different relationship between WC and skeletal appendicular mass, opposing our findings, that point to women with larger WC having lower values of lean mass [45]. However, the same individuals with higher WC values also showed worse muscle quality in physical performance tests, perhaps suggesting that the visceral adiposity index, measured in this study indirectly through WC, is negatively influencing muscle quality in individuals with metabolic syndrome [45].

In relation to the other adiposity indices, the different studies that used them had cardiovascular and metabolic diseases as their main evaluated outcomes [33–35], and the relationship between these indices and muscle mass has not been found to date.

According to the ROC curve analyzes performed, different cutoff points have been proposed to discriminate low muscle mass in middle-aged and older women. The cutoff point proposed for BMI was 29.4 kg/m^2^. However, this cutoff point differed from that proposed by Goodman et al. [46], who, using a sample of older participants in the NHANES study, found that BMI lower than 18 kg/m^2^ was related to the high probability (99%) of having low lean mass [46]. In a study developed by Keevil et al. [47], carried out with a heterogeneous sample of men and women between 48 and 92 years old, higher BMI values (> 29 kg/m^2^) were related to higher muscle strength values in the hand-grip exercise test, and inverse relationship was seen with the WC, as women with WC above 97 cm were those who obtained lower strength values, corroborating the cutoff point found in our study (94.3 cm) [47].

To date, no studies have been found that have sought to determine cutoff points for different adiposity indices, such as BAI, WHR, WHtR, CI and LAP that can predict low skeletal muscle mass. However, in the study conducted by Gadelha et al. [48], different cutoff points for adiposity indices were established to predict functional disability in older women (30-s-sit-up tests; 8-foot up-and-go test; and six-minute walk test). In that study, the cutoff point established for BMI, WC, WHR, CI, BAI were respectively: 26.93 (kg/m^2^), 89.5 (cm); 0.8 (cm/cm), 1.23 (AU) and 34.60 (5), values that are slightly lower than the values found in our study, this difference possibly being related to the difference in the characteristics inherent to the studied populations [48].

After logistic regression analysis, being over 60 years of age increases the chance of having low muscle mass by 3.8. This finding validates literature, since the progressive decrease in skeletal muscle mass increases with age [49] and occurs at a rate of 1.5% to 3% per year after the age of 60 [50]. In women, muscle tissue is sensitive to hormonal changes due to menopausal transition [5] and they have, on average, less muscle mass when compared to men [51]. In this context, the results of Sipila et al. [5] indicate that physical activity is particularly beneficial for women from middle age onward, emphasizing the importance of stimulating the performance of resistance training physical exercise, since it is capable of not only preventing the loss of muscle mass, but also increasing it [52], configuring the practice of physical activity as an important strategy during the aging process, especially in women.

Additionally, it was observed that WC above 94.3 increases the chance of having low muscle mass by 6.2. In the study by Abramowitz et al. [42], participants with low muscle mass had a higher WC – 115.6 (± 2.1) cm – when compared to those with preserved muscle mass – 109.5 (± 0.2) cm. Besides, in the longitudinal study by Kim [53], the changes resulting from the Muscle Mass Index over time were significantly associated with changes in WC, supporting our findings.

A similar relationship was observed in the other indices, where the cutoff points of 39.1 for BAI, WHR above 0.91, and WHtR above 0.64 increased the chance of women presenting low mass of about five times. The aforementioned indices have been proposed as viable alternatives for assessing obesity to the detriment of the limitations seen in classic measures such as BMI [54, 55]. However, literature still lacks evidence about the relationship between these indices and the muscle mass of middle-aged and older women.

Despite the lack of studies on the different adiposity and muscle mass indices, the relationship between obesity and skeletal muscle mass has already been established, and the repercussions on musculoskeletal performance are particularly important in women [56]. In this group, changes in skeletal muscle mass occur at earlier ages than men, that is, from middle age [57], as a consequence of the decline in estrogen resulting from the menopausal transition, culminating in direct repercussions in the increase of visceral fat [56], which when associated with reduced levels of physical activity, contributes to the increase in overweight and obesity, further expanding body changes [58].

These changes added to aging, in which changes in skeletal muscle tissue occur, such as reduction of muscle fibers, reduction of cross-sectional area, and reduction of the amount of muscle mass accompanied by deposition of intramuscular fat, lead to a decrease in the ability to generate strength and greater resistance to the anabolic stimulus [59, 60], which has serious repercussions on the functionality of this population.

Regarding the limitations of this study, the cross-sectional design limits causal inferences, which can be achieved only through longitudinal studies. For the calculation of skeletal muscle mass, bioimpedance was used. However, despite not being the gold standard, bioimpedance is widely used in research because it is portable, inexpensive, it does not expose individuals to radiation [29], and because it is considered a reliable method, in addition to having good correlation to the results found by Magnetic Resonance and Dual Emission X-Ray Densitometry [29, 61]. Another limitation refers to how we determined low muscle mass. In this study, we used the 20th percentile of the muscle mass distribution in the sample following previous studies [62, 63]. However, it is important to highlight that there are other ways to determine the cut-off for low-muscle mass described in the literature and the results may vary if different approaches are used [26].

As a strong point of this study, the anthropometric indices analyzed are well established in literature and were obtained through objective, valid, non-invasive and low-cost measures that can be easily accessible in clinical practice, including primary health care, allowing health practitioners to track women at risk of decreasing muscle mass and favoring early intervention, in order to prevent future adverse outcomes in these women’s functional health.

## Conclusions

Based on the exposed results, it can be concluded that after logistic regression analysis, age, WC, WHR, WHtR and BAI maintained an independent relationship with low muscle mass in middle-aged and older women. The present study pointed out the cutoff point of the anthropometric indices for the presence of low muscle mass. Among the anthropometric indices evaluated, BAI was the one with the best association with low muscle mass. Therefore, the present study points out that anthropometric indices can become effective and low-cost strategies to assess and manage health outcomes, which would be of great relevance in the aging process, especially in women, to whom body changes seem to be more striking. In addition, it would facilitate the improvement of actions aimed at this portion of the population, providing scientific support for the planning of prevention and health promotion actions.

## Data Availability

Datasets generated and analyzed during this study are available from the corresponding author on reasonable request.

## Abbreviations

BMI: Body mass index
WC: Waist circumference
WHR: Waist-to-hip ratio
WHtR: Waist-to-height ratio
CI: Conicity Index
VAI: Visceral adiposity index
LAP: Lipid accumulation product
HC: Hip circumference
BAI: Body adiposity index
TG: Triglycerides
HDL: High-density lipoprotein
mw: Appendicular skeletal muscle
MW: Minimum wage
OR: Odds ratio
